# Mining the stable quantitative trait loci for agronomic traits in wheat (*Triticum aestivum* L.) based on an introgression line population

**DOI:** 10.1186/s12870-020-02488-z

**Published:** 2020-06-15

**Authors:** Weiguo Chen, Daizhen Sun, Runzhi Li, Shuguang Wang, Yugang Shi, Wenjun Zhang, Ruilian Jing

**Affiliations:** 1 Shanxi Agricultural University, Taigu, 030801 Shanxi China; 2 Institute of Crop Science, Chinese Academy of Agricultural Sciences, Beijing, 100081 China

**Keywords:** Agronomic trait, Introgression line population, Quantitative trait loci, Wheat (*Triticum aestivum* L.)

## Abstract

**Background:**

Human demand for wheat will continue to increase together with the continuous global population growth. Agronomic traits in wheat are susceptible to environmental conditions. Therefore, in breeding practice, priority is given to QTLs of agronomic traits that can be stably detected across multiple environments and over many years.

**Results:**

In this study, QTL analysis was conducted for eight agronomic traits using an introgression line population across eight environments (drought stressed and well-watered) for 5 years. In total, 44 additive QTLs for the above agronomic traits were detected on 15 chromosomes. Among these, *qPH-6A*, *qHD-1A*, *qSL-2A*, *qHD-2D* and *qSL-6A* were detected across seven, six, five, five and four environments, respectively. The means in the phenotypic variation explained by these five QTLs were 12.26, 9.51, 7.77, 7.23, and 8.49%, respectively.

**Conclusions:**

We identified five stable QTLs, which includes *qPH-6A*, *qHD-1A*, *qSL-2A*, *qHD-2D* and *qSL-6A*. They play a critical role in wheat agronomic traits. One of the dwarf genes *Rht14*, *Rht16*, *Rht18* and *Rht25* on chromosome 6A might be the candidate gene for *qPH-6A*. The *qHD-1A* and *qHD-2D* were novel stable QTLs for heading date and they differed from known vernalization genes, photoperiod genes and earliness per se genes.

## Background

Wheat (*Triticum aestivum* L.) is a global food crop with an annual production of approximately 700 million tons [1]. Human demand for wheat will continue to increase together with the continuous global population growth. Wheat yield is associated with all major agronomic traits: for example, plant height affects dry matter accumulation and lodging resistance, spike length is the main factor that determines canopy distribution and influences light and CO_2_ utilization efficiency, and heading date (HD) plays a key role in the adaptation to various climates and cultivation methods [2]. The number of tillers, fertile spikelet number, grain number per spike, thousand-grain weight, and grain weight per plant also directly determine the yield per unit area of wheat. For these reasons, it is necessary to understand the genetic mechanisms that underlie agronomic traits in wheat, to improve its yield. However, agronomic traits in wheat are susceptible to environmental conditions, because they are mostly quantitative traits controlled by minor genes. Therefore, it is imperative to identify elite alleles across multiple environments to improve existing wheat cultivars [3].

Introgression line (IL) populations provide excellent material for QTL mapping. Currently, it is difficult to fine-map QTLs using conventional QTL mapping populations, due to their complex genetic background. By contrast, QTL mapping using IL populations can eliminate interference from the genetic background and improve the accuracy of gene mapping. Furthermore, fine mapping of the target QTLs can be achieved by constructing secondary segregating populations. In addition, IL populations play a pivotal role in the pyramid breeding of elite alleles. To date, many QTLs for agronomic traits in wheat have been detected using IL populations. For example, Pestsova et al. [4] identified seventeen significant QTLs for agronomic traits using an IL population derived from the substitution lines ‘Chinese Spring’/‘Synthetic 6x’. Furthermore, Huang et al. [5, 6] analyzed QTLs related to agronomic traits with two IL populations derived from ‘Prinz’/‘W-7984’ and ‘Flair’/‘XX86’, respectively, and Yan et al. [7] mapped QTLs for ten agronomic traits using 160 BC_3_F_3_ ILs derived from a cross between Lumai14 and Jing411. Ibrahim et al. [8] used the ‘Triso’/‘Syn084’ IL population to map QTLs and identified seven QTLs for heading date, five QTLs for days to maturity, three QTLs for number of spikes per plant, six QTLs for thousand grain weight, and seven QTLs for grain yield. However, quantitative traits are extremely susceptible to environmental conditions and a high probability of interactions between genes and the environment exists. Therefore, in breeding practice, priority is given to QTLs of agronomic traits that can be stably detected across multiple environments and over many years.

The objective of this study was to identify stable QTLs for agronomic traits in wheat across multiple environments. Therefore, an IL population was constructed between the wheat cultivars Lumai 14 (recurrent parent) and Shaanhan 8675, and agronomic traits were then mapped by QTL analysis using the IL population across eight environments for 5 years. The results are relevant for the subsequent construction of near-isogenic lines and for the fine mapping and cloning of QTLs and marker-assisted selection of high-yield wheat.

## Results

### Phenotypic analysis

In this study, we analyzed eight agronomic traits in the two parents and the IL population. The results revealed that Shaanhan 8675 was a high-value parent, whereas Lumai 14 was a low-value parent in terms of plant height, spike length, the number of valid tillers, fertile spikelet number per main spike, grain weight per plant, thousand-grain weight, and grain number per spike. Each of the seven traits differed significantly (*P <* 0.05 or 0.01) in at least two environments. By contrast, heading date exhibited an inverse trend. Specifically, Lumai 14 was a high-value parent with late heading, whereas Shaanhan 8675 was a low-value parent with early heading. The heading date of the two parents differed significantly in three environments, E4 (*P <* 0.05), E5 (*P <* 0.05), and E6 (*P <* 0.01). In the IL population, all eight agronomic traits showed continuous variation over a particularly large range in each environment. In most cases, the trait values of the IL population presented a normal distribution and showed bidirectional transgressive segregation (Additional file 1).

### Detection of additive QTLs

**Fig. 1:**
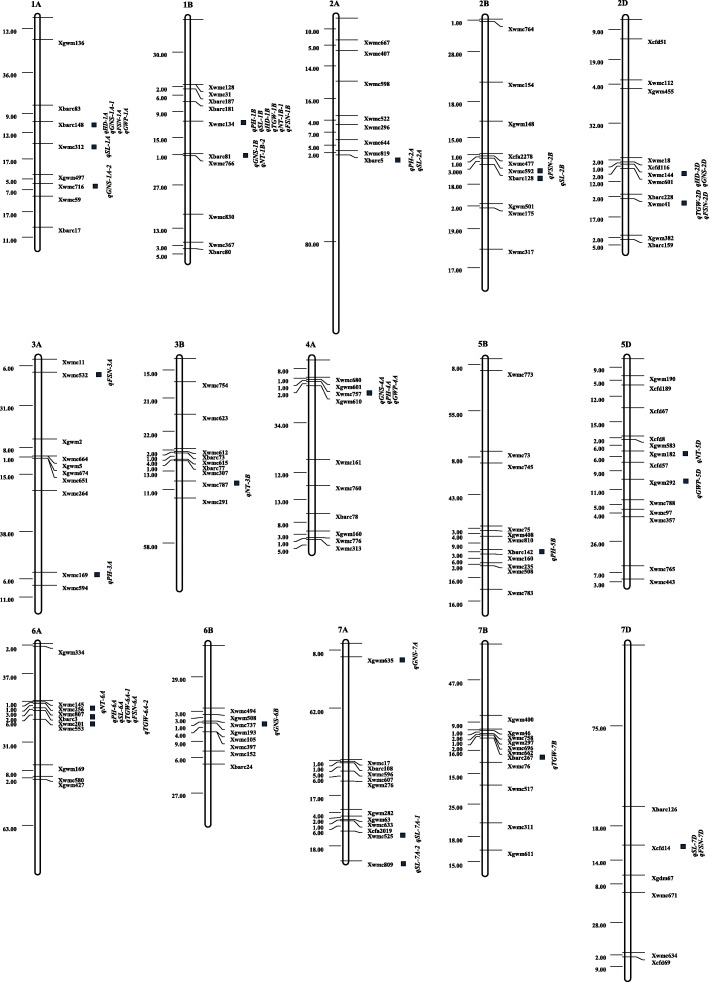
Linkage maps showing the positions of QTLs for important agronomic traits mapped in a Lumai 14 × Shaanhan 8675 IL population

Using the IL population derived from Lumai 14 × Shaanhan 8675, we detected a total of 44 additive QTLs for agronomic traits in wheat (Table 1; Fig. 1). These QTLs were distributed on chromosomes 1A, 2A, 3A, 4A, 6A, 7A, 1B, 2B, 3B, 5B, 6B, 7B, 2D, 5D and 7D. Their logarithm of odds (LOD) varied from 2.50 to 12.91, and the phenotypic variance explained (PVE) by the QTLs ranged between 1.33 and 22.70%. The number of QTLs that controlled plant height, spike length, heading date, grain number per spike, thousand-grain weight, the number of valid tillers, fertile spikelet number per main spike, and grain weight per plant, was six, eight, three, seven, five, five, seven, and three, respectively. Among them, 15 QTLs had positive alleles from the recipient parent Lumai 14, whereas the remaining 29 QTLs had positive alleles from the donor parent Shaanhan 8675.

**Table 1:** Additive effect QTLs for important agronomic traits in a Lumai 14 × Shaanhan 8675 IL population

Trait	QTL	Environment	Marker	LOD	Additive effect^a^	PVE (%)^b^
PH	*qPH-1B*	E2	Xwmc134	3.31	−1.09	7.08
	*qPH-2A*	E8	Xbarc5	2.56	−1.75	3.36
	*qPH-3A*	E8	Xwmc169	6.04	8.71	8.34
	*qPH-4A*	E5	Xwmc757	5.22	−2.30	2.05
	*qPH-5B*	E5	Xbarc142	3.46	4.79	1.33
	*qPH-6A*	E1, E2, E4, E5, E6, E7, E8	Xbarc3	6.99, 6.24, 7.13, 8.87, 12.91, 9.90, 6.16	2.27, 1.61, 2.19, 2.57, 2.35, 1.98, 2.32	17.18, 12.21, 3.22, 3.81, 22.70, 17.87, 8.81
SL	*qSL-1A*	E1, E3, E7	Xwmc312	2.96, 5.11, 3.62	0.20, 0.25, 0.23	6.38, 10.26, 7.89
	*qSL-1B*	E2, E5	Xwmc134	3.41, 2.92	−0.21, − 0.18	8.70, 5.13
	*qSL-2A*	E3, E4, E5, E6, E8	Xbarc5	3.56, 3.70, 6.52, 2.53, 5.78	−0.25, − 0.28, − 0.34, − 0.23, − 0.35	7.07, 6.74, 9.84, 4.76, 10.44
	*qSL-2B*	E5	Xbarc128	4.35	1.21	6.49
	*qSL-6A*	E1, E4, E6, E8	Xbarc3	4.47, 3.45, 3.89, 5.41	0.24, 0.22, 0.24, 0.28	10.00, 6.35, 7.59, 10.01
	*qSL-7A-1*	E3	Xwmc525	3.57	0.8	7.20
	*qSL-7A-2*	E1	Xwmc809	2.72	0.68	5.86
	*qSL-7D*	E4, E5, E8	Xcfd14	2.54, 4.02, 2.86	0.20, 0.22, 0.21	4.55, 5.86, 4.95
HD	*qHD-1A*	E1, E2, E4, E5, E6, E8	Xbarc148	4.32, 3.39, 10.04, 9.49, 6.42, 3.85	−0.69, −0.50, −0.82, −0.73, −0.53, − 0.51	8.43, 7.07, 11.34, 12.42, 11.05, 6.76
	*qHD-1B*	E5	Xwmc134	6.49	0.42	8.62
	*qHD-2D*	E4, E5, E6, E7, E8	Xwmc144	4.19, 5.37, 6.48, 4.72, 2.99	−0.53, −0.56, − 0.55, − 0.65, − 0.47	5.21, 8.50, 11.09, 6.43, 4.93
GNS	*qGNS-1A-1*	E2	Xbarc148	5.48	3.29	8.80
	*qGNS-1A-2*	E4	Xwmc716	5.73	9.60	5.46
	*qGNS-1B*	E6	Xbarc81	2.74	1.71	3.10
	*qGNS-2D*	E3, E4, E5	Xwmc144	3.29, 4.21, 2.67	−2.05, −2.08, −1.68	6.47, 4.09, 3.96
	*qGNS-4A*	E4	Xwmc757	2.60	−1.45	2.44
	*qGNS-6B*	E3	Xwmc737	2.79	7.18	5.14
	*qGNS-7A*	E8	Xgwm635	4.21	2.64	6.86
TGW	*qTGW-1B*	E3	Xwmc134	2.72	−1.17	5.65
	*qTGW-2D*	E4	Xwmc41	3.17	0.86	3.26
	*qTGW-6A-1*	E4, E8	Xbarc3	4.00, 3.19	0.89, 1.05	4.05, 4.28
	*qTGW-6A-2*	E5, E8	Xwmc201	7.97, 6.19	1.66, 1.40	12.13, 8.46
	*qTGW-7B*	E8	Xbarc267	2.57	−1.81	3.30
NT	*qNT-1B-1*	E1	Xwmc134	2.64	0.21	5.19
	*qNT-1B-2*	E5	Xbarc81	3.43	0.25	4.13
	*qNT-3B*	E4	Xwmc787	3.01	0.67	3.19
	*qNT-5D*	E6	Xgwm182	2.70	0.40	3.62
	*qNT-6A*	E3, E7	Xwmc256	5.29, 4.22	−0.43, −0.21	7.15, 4.95
FSN	*qFSN-1A*	E8	Xbarc148	4.58	−0.31	5.46
	*qFSN-1B*	E7	Xwmc134	2.87	0.22	4.59
	*qFSN-2B*	E8	Xwmc592	5.75	1.49	7.14
	*qFSN-2D*	E4	Xwmc41	2.50	0.22	2.56
	*qFSN-3A*	E5	Xwmc532	6.99	1.65	6.72
	*qFSN-6A*	E3	Xbarc3	2.72	−0.26	5.24
	*qFSN-7D*	E1, E4, E8	Xcfd14	2.79, 3.69, 7.39	0.30, 0.25, 0.33	5.43, 3.46, 9.16
GWP	*qGWP-1A*	E2	Xbarc148	4.21	0.41	6.73
	*qGWP-4A*	E4	Xwmc757	4.89	−0.40	4.44
	*qGWP-5D*	E3	Xgwm292	2.89	0.95	4.51

^a^Positive values indicate that ‘Shanhan 8675’ alleles increase the corresponding trait, and, conversely, negative values indicate that ‘Shanhan 8675’ alleles decrease it

^b^Phenotypic variance explained by the additive QTL

Five additive QTLs (*qPH-6A*, *qHD-1A*, *qSL-2A*, *qHD-2D*, *qSL-6A*) were detected in four or more environments. Four of these: *qPH-6A*, *qHD-1A*, *qSL-2A*, and *qHD-2D*, were not only studied in well-watered conditions but also in drought-stress environment, whereas *qSL-6A* for spike length was only detected in four drought-stress environments. Additionally, *qPH-6A* and *qSL-6A* had positive additive effects and their positive alleles were derived from Shaanhan 8675, but *qSL-2A*, *qHD-1A* and *qHD-2D* had negative additive effects and their positive alleles were derived from Lumai 14.

### Genetic effects of stable QTLs

In total, 38 lines contained introgressed segments from the donor parent at the *qPH-6A* locus (Additional file 2). Among these, 21 lines that were introgressed with only *qPH-6A* (without other QTLs for plant height) exhibited positive effects for plant height in the eight environments (E1 to E8), with means of 7.23, 2.76, − 1.71%, 6.16, 6.52, 7.02, 6.82, and 0.93%, respectively. Their mean positive effects in five drought-stress conditions was 4.82% and the mean positive effect in three well-watered conditions was 3.88%.

Out of 23 lines that contained introgressed segments from the donor parent at the *qSL-2A* locus (Additional file 3), three lines that were introgressed with only *qSL-2A* (without other QTLs for spike length) exhibited negative effects for spike length in the eight environments (E1 to E8), with means of 3.49, 2.72, 11.19, 8.71, 8.69, 10.03, 3.36, and 13.92%, respectively. Their mean negative effect in five drought-stress conditions was 7.78%, and the mean negative effect in three well-watered conditions was 7.75%. In particular, line 39 contained only seven introgressed donor segments, and except for *qSL-2A*, no other QTLs for spike length were detected. However, this line exhibited a significant difference in spike length (*P <* 0.05 or 0.01) compared with Lumai 14 across six environments.

Twenty lines contained introgressed segments from the donor parent at the *qHD-1A* locus (Additional file 4). Among these, 11 lines that were introgressed with only *qHD-1A* (without other QTLs for heading date) showed an earlier heading date by 0.75, 1.20, − 0.21, 1.12, 2.24, 1.31, 1.12, and 1.36 days on average across the eight environments (E1 to E8), respectively. Heading date was on average 1.15 and 1.05 days earlier in the five drought-stress conditions and three well-watered conditions, respectively.

Out of the 18 lines that contained introgressed segments from the donor parent at the *qHD-2D* locus, (Additional file 5), seven lines introgressed with only *qHD-2D* (without other QTLs for heading date) advanced heading date by 0.32, 0.41, 0.00, -0.14, 1.52, 1.25, 1.86, and 1.54 days on average across the eight environments (E1 to E8), respectively. Heading date was advanced by an average of 0.68 days in five drought-stress conditions and 1.13 days in three well-watered conditions.

The *qSL-6*A QTL was only detected in four drought-stress conditions (E1, E4, E6, and E8). Out of 38 lines that contained introgressed segments from the donor parent at this locus (Additional file 6), 16 lines that were introgressed with only *qSL-6A* (without other QTLs for spike length) exhibited positive effects for spike length in five drought-stress environments (E1, E2, E4, E6, and E8), with means of 5.37, 1.40, 2.52, 1.36, and 2.65%, respectively. Their mean positive effect in five drought-stress conditions was 2.66%. In particular, line 152 (with *qSL-6A* and *qSL-7D* from the donor parent) exhibited a significant difference in spike length (*P <* 0.01) compared with Lumai 14 across eight environments.

## Discussion

In this study, all of the eight studied agronomic traits are quantitatively inherited. Because the stable QTLs related to these traits should be repeatedly detected in multiple environments, we performed a QTL analysis for the eight agronomic traits using the IL population across 5 years. In total, forty-four QTLs for the agronomic traits were identified. Among these, some QTLs were identical to those detected in previous studies. For example, *qTGW-2D* for thousand-grain weight was the same marker locus as *QTgw.nfcri-2D* and *QTkw.ncl-2D.2*, which are QTLs for thousand-grain weight reported by Wang et al. [9] and Ramya et al. [10], respectively. The *qGNS-6B* QTL for grain number per spike was located within the same marker region as *QKNPS-DH-6B*, which affects kernel number per spike and was detected by Zhang et al. [11]. We mapped a QTL controlling plant height, *qPH-6A* near the Xbarc3. Similarly, Buerstmayr et al. [12] also detected a QTL for plant height in the Xs18m24_8-Xbarc3 interval. We detected *qFSN-7D* in three different environments. Ma et al. [13] also detected QTLs for spikelet number per spike and fertile spikelet number at the same location. We detected *qTGW-6A-1* on chromosome 6A, which controlled thousand-grain weight in two environments, with a mean PVE of 4.17%. Wang et al. [14] also detected the QTL for thousand-grain weight in the Xbarc3–XwPt-5094 interval across three environments, with a mean PVE of 9.4%.

Furthermore, some QTLs were found in multiple environments; for example, *qPH-6A* was detected in seven environments (E1, E2, E4, E5, E6, E7, and E8), *qHD-1A* in six environments (E1, E2, E4, E5, E6, and E8), *qSL-2A* in five environments (E3, E4, E5, E6, and E8), *qHD-2D* in five environments (E4, E5, E6, E7, and E8), and *qSL-6A* in four drought stress environments, E1, E4, E6, and E8. Based on genetic effects (Additional files 2, 3, 4, 5, 6), these stable QTLs all played important roles in the development of certain agronomic traits. Additionally, the stability of these QTLs across multiple environments was important for motivating further interest in molecular mechanism studies of agronomic trait development. So, the stable QTLs detected in this study are preferential genes for fine mapping and marker-assisted selection of wheat in the future. Particularly, priority should be given to those QTLs with large or pleiotropic genetic effects that have not been applied in breeding [15]. In addition, 31 QTLs were only detected in one environment in this study, but it was difficult for them to be applied in breeding, due to their relative sensitivity to environmental conditions.

By contrast, we found that the spike length of line 152 (with *qSL-6A* and *qSL-7D* from Shaanhan8675) was significantly longer than that of the recurrent parent ‘Lumai 14’ in all eight environments (Additional file 6). In addition, the spike length of line 39 (with *qSL-2A* from Shaanhan8675) was significantly smaller than that of the recurrent parent ‘Lumai 14’ in six environments. Seven introgressed donor chromosomal segments were carried by line 39 (Additional file 3), but only *qSL-2A* controlled spike length. Therefore, line 152 and line 39 are thought to be the potential near-isogenic lines that can be used to fine-map and clone *qSL-2A* and *qSL-6A.*

To date, the dwarf genes identified on chromosome 6A include: *Rht14*, *Rht16*, *Rht18*, *Rht24*, and *Rht25*. The *Rht24* locus is located between the Xbarc103 and Xwmc256 markers [16] and the remaining four genes are close to the Xbarc3 marker [17–20]. Haque et al. [17] thought *Rht14* was allelic to *Rht16* and *Rht18* and linked to SSR marker Xbarc3 on chromosome 6AS. However, Vikhe et al. [18] suggested that *Rht14* might not be allelic to *Rht18*. Moreover, in addition to reducing plant height, it was found that *Rht18* affected spikelet number per spike, grain number per spike, and thousand-grain weight [20]. *Rht25* also significant affected spike length, heading date, spikelet number per spike, spikelet density, grain number per spike and grain weight [19]. In the study, a major QTL for plant height, *qPH-6A* was also detected near the Xbarc3 across seven environments, with a PVE of 3.81–22.70% (mean 12.26%). And it was found that the QTL was also pleiotropic and was responsible for plant height, spike length, thousand-grain weight and fertile spikelet number per main spike in wheat. Therefore, we thought one of the dwarf genes located on chromosome 6A might be the candidate gene for *qPH-6A*. Exactly which dwarf gene plays a role needs to be further analyzed.

Heading date is mainly regulated by three types of genes in wheat; namely, those involved in vernalization (*Vrn*), photoperiod (*Ppd*), and earliness per se (*Eps*). The genes that regulate vernalization in wheat include *Vrn-1*, *Vrn-2*, *Vrn-3*, and *Vrn-4* [21, 22]. The three orthologous genes of *Vrn-1*, *Vrn-A1*, *Vrn-B1*, and *Vrn-D1*, located on chromosomes 5A, 5B, and 5D, respectively. *Vrn-A2* was also mapped to chromosome 5A of *Triticum monococcum* [23], whereas the three orthologous genes of *Vrn-3, Vrn-A3*, *Vrn-B3*, and *Vrn-D3*, were distributed on chromosomes 7A, 7B, and 7D, respectively [24–26]. The *Vrn-4* gene (designated *Vrn-D4*) was mapped to chromosome 5D of hexaploid spring wheat [27]. For *Ppd* genes, *Ppd-A1*, *Ppd-B1*, *Ppd-D1*, and *Ppd-B2* had been cloned and were respectively mapped to chromosomes 2A, 2B, 2D, and 7BS [28–30]. Here, a stable QTL for heading date, *qHD-2D*, was also mapped on chromosome 2D. Comparative analysis found that *Ppd-D1* was located on 2DS, while *qHD-2D* was on 2DL. So, they are not the same locus. The *Eps* genes/QTLs are reported to be present on all wheat chromosomes [27]. Among them, *Eps-A*^*m*^*1* from *T. monococcum* was located close to the Xwg241 on 1AL [31]. In the study, another stable QTL for heading date, *qHD-1A*, was detected on 1AS across multiple environments. Clearly, the two are not the same locus. Given the high stability of *qHD-1A* and *qHD-2D* in this study, we consider it is important to study these two QTLs further and to apply them to breeding programmes.

QTL hotspots indicate the location of a single QTL with pleiotropic effect or tightly linked QTLs [32, 33]. In this study, we detected some QTL hotspots on chromosomes 1A, 1B, 2A, 2D, 4A, and 6A. Four QTLs were detected near the Xbarc148 marker on chromosome 1A, which individually controlled heading date, grain number per spike, fertile spikelet number per main spike, or grain weight per plant. Six QTLs were detected near the Xwmc134 marker on chromosome 1B and affected plant height, spike length, heading date, thousand-grain weight, the number of valid tillers, and fertile spikelet number per main spike. Two QTLs associated with grain number per spike and the number of valid tillers, respectively, were detected near Xbarc81 on chromosome 1B. Two QTLs related to plant height and spike length, respectively, were detected near Xbarc5 on chromosome 2A. Two QTLs controlling different traits were also detected near each of the Xwmc144 and Xwmc41 markers on 2D chromosome. In addition, three QTLs that controlled plant height, grain number per spike and grain weight per plant were detected near Xwmc757 on chromosome 4A. Moreover, six QTLs were detected between Xwmc145 and Xwmc553 (13 cM) on chromosome 6A, which affected the number of valid tillers, plant height, spike length, thousand-grain weight and fertile spikelet number per main spike, respectively. It is worth noting that all of the stable QTLs detected in this study were located in the “QTL-hotspot” regions, which highlights the importance of these regions in the development of agronomic traits in wheat. These stable QTLs in the “QTL-hotspot” regions could be ideal for understanding their regulatory roles. Therefore, these “QTL-hotspot” regions may be the most important targets for breeding of agronomic traits. We can pyramid positive alleles of these QTLs to synergistically improve their genetic effects and substantially increase genetic improvement efficiency in wheat.

## Conclusions

In our study, five stable QTLs—*qPH-6A*, *qHD-1A*, *qSL-2A*, *qHD-2D* and *qSL-6A—*were identified using an introgression line population across eight environments (drought-stressed and well-watered) for 5 years. All the stable QTLs were located within “QTL-hotspot” regions and played critical roles in agronomic traits of wheat. *qPH-6A* was pleiotropic; it played an important effect on plant height, and its effects on spike length, thousand-grain weight, and fertile spikelet number per main spike were also found. The candidate gene for *qPH-6A* might be one of the dwarf genes (*Rht14*, *Rht16*, *Rht18* and *Rht25*) on chromosome 6A. *qHD-1A* and *qHD-2D* were both novel stable QTLs affecting heading date and they differed from known vernalization, photoperiod and earliness per se genes. Both *qSL-2A* and *qSL-6A* controlled spike length; the effect of *qSL-2A* was seen in both well-watered and drought-stress conditions, whereas the effect of *qSL-6A* was seen only in the drought-stress condition. These results are relevant to gene fine mapping and cloning, as well as marker-assisted selection of high-yield wheat.

## Methods

### Plant material

**Fig. 2:**
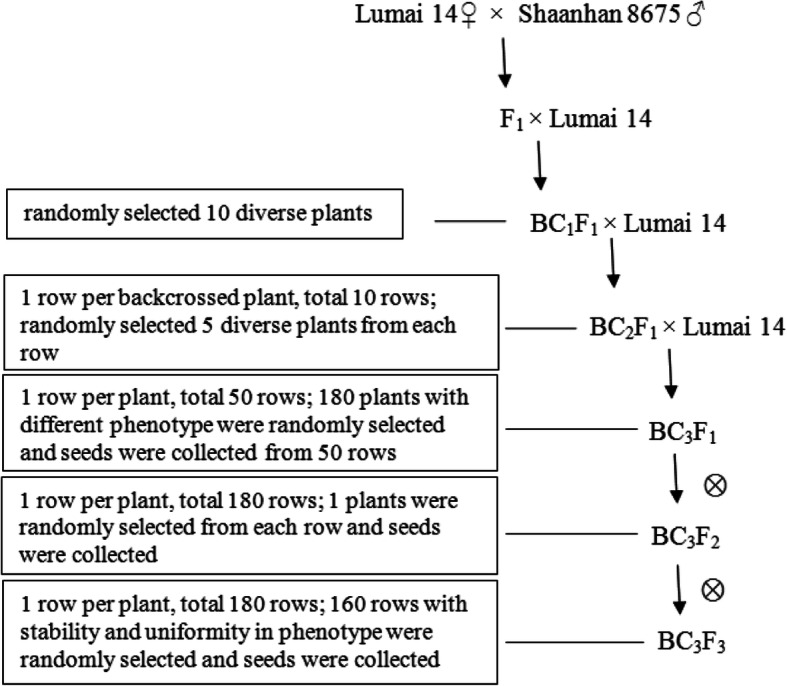
Flowchart for the construction of an introgression line population derived from wheat cultivars Lumai 14 and Shaanhan 8675

An IL population (BC_3_F_3_–BC_3_F_7_) of wheat was derived from two wheat cultivars Lumai 14 and Shaanhan 8675. Lumai 14 was the recipient and recurrent parent, and Shaanhan 8675 was the donor parent. Both materials were legally obtained from the National Genebank of China. The IL population were built by the co-author Ruilian Jing, a professor of the Institute of Crop Science, Chinese Academy of Agricultural Sciences (Beijing, China). The population consisted of 160 lines, with Lumai 14 as the recurrent parent and Shaanhan 8675 as the donor parent (Fig. 2). Each line contained eight segments from the donor on average. Only one donor segment was introgressed into IL-1, with a length of 12.8 cM, whereas the greatest number of donor fragments (i.e., 46) was introgressed into IL-45, with a total length of 940.3 cM.

### Field experiments

The IL population and its parents were grown in the wheat experimental field of Shanxi Agricultural University (Taigu, China) during the growing seasons of 2010–2011 (BC_3_F_3_), 2011–2012 (BC_3_F_4_), 2013–2014 (BC_3_F_5_), 2014–2015 (BC_3_F_6_), and 2015–2016 (BC_3_F_7_). They were planted in eight environments; namely drought stress in 2010–2011 (E1), drought stress in 2011–2012 (E2), well-watered conditions in 2011–2012 (E3), drought stress in 2013–2014 (E4), well-watered conditions in 2013–2014 (E5), drought stress in 2014–2015 (E6), well-watered conditions in 2014–2015 (E7), and drought stress in in 2015–2016 (E8).

After sowing, no irrigation was applied to plants in the drought-stress environments throughout the wheat growth period. The precipitation during the wheat growth period was 110 mm in 2010–2011, 197 mm in 2011–2012, 185 mm in 2013–2014, 109 mm in 2014–2015, and 218 mm in 2015–2016. Plants grown in the well-watered environments were irrigated before wintering, at jointing, and in the mid-stage of grain filling, with 650 m^3^/hm^2^ water each time. The treatments were arranged in a random block design, with three replicates. Each plot contained 17 rows, with a row length of 2.5 m and a row spacing of 0.25 m. Sixty seeds were dibbled per row.

### Phenotyping

The date of heading was recorded at the stage when half of the spikes had emerged from the flag leaf sheath in more than 50% of the plants of each line. The heading date (HD) was calculated by subtracting the date of heading by the date of sowing. Ten plants with robust and uniform growth were selected at random from each line to measure plant height (PH) and spike length (SL) at the milky ripe stage. After harvest, the number of valid tillers (NT), fertile spikelet number per main spike (FSN), grain number per spike (GNS), grain weight per plant (GWP), and thousand-grain weight (TGW) were determined in the laboratory.

### Genetic map construction

Extraction of DNA was performed using the CTAB method. Following the principle of uniform distribution on the chromosome, 565 simple sequence repeat (SSR) markers covering the whole genome of wheat were selected based on the high-density microsatellite consensus map [34]. The selected SSR markers were screened for polymorphisms between the two parents, which yielded 187 polymorphic markers. The polymorphic SSR markers were then used to perform a genome-wide screen of the IL population. In addition, the polymorphic SSR markers were used to generate a genetic linkage map using Map Draw software [35], based on the high-density microsatellite consensus map [34]. In the map, recombination distances were determined using the Kosambi mapping function. The total length of the map was 2569 cM and the mean distance between the markers was 13.73 cM.

### Data analysis

The phenotypic data for agronomic traits were analyzed using SPSS 13.0 (SPSS Inc., Chicago, USA) and Excel 2007 (Microsoft Corp., Redmond, USA). QTL detection was performed using ICIMapping v4.0 (http://www.isbreeding.net/) via a likelihood ratio test based on stepwise regression (RSTEP-LRT; Permutation times = 1000, *P* < 0.05). QTLs were described by the nomenclature *q trait-chromosome*.

## Supplementary information

**Additional file 1.** Phenotypic values and distribution parameters for agronomic traits of parents and introgression lines.

**Additional file 2. **Plant height characteristics in wheat lines carrying introgressed donor chromosomal segments at the *qPH-6A* locus.

**Additional file 3. **Spike length characteristics in wheat lines carrying introgressed donor chromosomal segments at the *qSL-2A* locus.

**Additional file 4. **Heading date characteristics in wheat lines carrying introgressed donor chromosomal segments at the *qHD-1A* locus.

**Additional file 5. **Heading date characteristics in wheat lines carrying introgressed donor chromosomal segments at the *qHD-2D* locus.

**Additional file 6. **Spike length characteristics in wheat lines carrying introgressed donor chromosomal segments at the *qSL-6A* locus.

## Data Availability

All data generated in this study are included in the paper and the supporting information files. The datasets of genetic polymorphisms from all lines of ‘Lumai 14’ × ‘Shaanhan 8675’ IL population are available from the Figshare Digital Repository: 10.6084/m9.figshare.12442112.v1.

## Abbreviations

QTLs: Quantitative trait loci
IL: Introgression line
PH: Plant height
SL: Spike length
NT: The number of valid tillers
FSN: Fertile spikelet number per main spike
GNS: Grain number per spike
GWP: Grain weight per plant
TGW: Thousand-grain weight
HD: Heading date
