# Supplementary material for: Mining the stable quantitative trait loci for agronomic traits in wheat (Triticum aestivum L.) based on an introgression line population
Source: BMC Plant Biol. 2020 Jun 15;20:275. doi: 10.1186/s12870-020-02488-z (PMC7296640; doi:10.1186/s12870-020-02488-z)
Supplement: Supplementary file 1 — Additional file 1. Phenotypic values and distribution parameters for agronomic traits of parents and introgression lines. [file 12870_2020_2488_MOESM1_ESM.docx]

**Additional file 1** Phenotypic values and distribution parameters for agronomic traits of parents and introgression lines

| Trait | Environment | Parent | | | |  | | Introgression Lines | | | | | | | | |
| --- | --- | --- | --- | --- | --- | --- | --- | --- | --- | --- | --- | --- | --- | --- | --- | --- |
|  |  | Lumai14 | | Shaanhan 8675 | |  | | Mean | | SD | Variation | Skewness | | Kurtosis | | CV (%) |
| PH | E1 | 53.76 | 60.68 | |  | | 55.90 | | 4.50 | | 44.92–71.71 | 0.53 | 0.19 | | 8.05 | |
|  | E2 | 52.65 | 59.85^*^ | |  | | 51.16 | | 3.51 | | 39.83–61.63 | -0.24 | 0.62 | | 6.86 | |
|  | E3 | 56.38 | 65.95 | |  | | 53.93 | | 4.37 | | 38.13–67.50 | -0.20 | 0.94 | | 8.10 | |
|  | E4 | 60.98 | 76.78^**^ | |  | | 61.36 | | 4.23 | | 47.59–79.12 | 0.34 | 1.81 | | 6.89 | |
|  | E5 | 77.27 | 95.81^**^ | |  | | 78.36 | | 5.44 | | 63.43–100.01 | 0.24 | 1.53 | | 6.94 | |
|  | E6 | 51.83 | 61.90^*^ | |  | | 52.44 | | 3.58 | | 43.64–62.63 | 0.22 | 0.24 | | 6.83 | |
|  | E7 | 52.43 | 61.57 | |  | | 53.72 | | 3.44 | | 45.57–66.33 | 0.58 | 1.33 | | 6.40 | |
|  | E8 | 59.83 | 69.04 | |  | | 57.70 | | 5.38 | | 45.47–79.47 | 0.90 | 1.90 | | 9.32 | |
| SL | E1 | 7.54 | 8.28 | |  | | 7.70 | | 0.62 | | 6.22–9.51 | 0.25 | 0.48 | | 8.05 | |
|  | E2 | 8.21 | 8.60 | |  | | 8.26 | | 0.65 | | 6.33–9.83 | -0.28 | 0.34 | | 7.87 | |
|  | E3 | 8.52 | 8.87 | |  | | 8.09 | | 0.62 | | 6.42–9.80 | -0.23 | 0.24 | | 7.66 | |
|  | E4 | 8.90 | 9.98^**^ | |  | | 8.86 | | 0.66 | | 6.70–11.05 | -0.43 | 0.67 | | 7.45 | |
|  | E5 | 9.09 | 9.34 | |  | | 9.08 | | 0.65 | | 7.57–10.83 | -0.15 | 0.01 | | 7.16 | |
|  | E6 | 8.81 | 9.42 | |  | | 8.50 | | 0.65 | | 7.01–10.27 | -0.17 | 0.01 | | 7.65 | |
|  | E7 | 8.50 | 9.63^*^ | |  | | 8.53 | | 0.62 | | 6.84–10.13 | -0.24 | -0.00 | | 7.27 | |
|  | E8 | 8.24 | 8.47 | |  | | 7.99 | | 0.68 | | 6.41–10.23 | -0.03 | 0.78 | | 8.51 | |
| HD | E1 | 224.84 | 224.83 | |  | | 225.08 | | 1.34 | | 222.00–229.50 | 0.44 | 0.30 | | 0.60 | |
|  | E2 | 224.17 | 223.34 | |  | | 223.97 | | 1.08 | | 222.00–227.50 | 0.84 | 1.00 | | 0.48 | |
|  | E3 | 223.00 | 222.67 | |  | | 223.68 | | 1.11 | | 220.00–227.50 | 0.07 | 2.24 | | 0.50 | |
|  | E4 | 216.00 | 213.56^*^ | |  | | 216.66 | | 1.15 | | 213.67–219.00 | -0.53 | -0.20 | | 0.53 | |
|  | E5 | 218.33 | 214.67^*^ | |  | | 217.31 | | 1.13 | | 214.67–220.00 | -0.01 | -0.68 | | 0.52 | |
|  | E6 | 216.11 | 214.11^**^ | |  | | 215.78 | | 0.97 | | 213.33–218.33 | 0.10 | -0.27 | | 0.45 | |
|  | E7 | 219.67 | 217.33 | |  | | 219.13 | | 1.15 | | 216.33–221.33 | -0.01 | -0.57 | | 0.52 | |
|  | E8 | 222.78 | 221.33 | |  | | 222.26 | | 1.10 | | 220.00–227.00 | 0.84 | 1.27 | | 0.49 | |
| GNS | E1 | 34.65 | 36.35 | |  | | 31.62 | | 3.69 | | 20.85–41.70 | 0.15 | 0.40 | | 11.67 | |
|  | E2 | 30.68 | 36.30 | |  | | 34.70 | | 5.71 | | 15.36–48.55 | -0.03 | 0.29 | | 16.46 | |
|  | E3 | 37.09 | 46.33^**^ | |  | | 36.81 | | 4.33 | | 23.60–51.50 | -0.10 | 0.47 | | 11.76 | |
|  | E4 | 34.55 | 42.99^**^ | |  | | 32.82 | | 3.98 | | 19.40–41.33 | -0.31 | 0.17 | | 12.13 | |
|  | E5 | 30.65 | 41.27^**^ | |  | | 39.15 | | 3.93 | | 26.79–49.53 | -0.07 | 0.62 | | 10.04 | |
|  | E6 | 37.17 | 40.54 | |  | | 34.34 | | 4.17 | | 24.32–43.84 | -0.22 | 0.38 | | 12.14 | |
|  | E7 | 43.41 | 50.09 | |  | | 40.38 | | 4.58 | | 26.33–55.71 | 0.23 | -0.65 | | 11.34 | |
|  | E8 | 39.50 | 39.47 | |  | | 37.89 | | 5.38 | | 24.83–52.57 | 0.14 | -0.46 | | 14.20 | |
| TGW | E1 | 39.22 | 41.80 | |  | | 36.42 | | 4.82 | | 23.38–48.42 | -0.01 | -0.21 | | 13.23 | |
|  | E2 | 37.40 | 36.59 | |  | | 38.88 | | 4.07 | | 25.80–53.37 | 0.24 | 1.15 | | 10.47 | |
|  | E3 | 42.44 | 47.15 | |  | | 42.90 | | 3.88 | | 30.77–55.33 | 0.05 | 0.30 | | 9.04 | |
|  | E4 | 34.19 | 37.03^*^ | |  | | 37.17 | | 2.45 | | 30.08–45.03 | 0.17 | 0.10 | | 6.59 | |
|  | E5 | 38.69 | 44.63^*^ | |  | | 42.45 | | 3.25 | | 34.27–50.87 | 0.14 | -0.36 | | 7.66 | |
|  | E6 | 36.98 | 37.72 | |  | | 40.28 | | 2.77 | | 32.30–46.96 | -0.06 | 0.16 | | 6.88 | |
|  | E7 | 36.92 | 37.14 | |  | | 38.52 | | 3.05 | | 31.49–48.33 | 0.26 | 0.36 | | 7.92 | |
|  | E8 | 40.33 | 42.23 | |  | | 41.67 | | 3.53 | | 32.85–50.00 | -0.12 | -0.19 | | 8.47 | |
| NT | E1 | 2.78 | 3.28 | |  | | 3.65 | | 0.71 | | 1.55–5.45 | 0.16 | -0.02 | | 19.45 | |
|  | E2 | 1.73 | 2.28 | |  | | 1.75 | | 0.54 | | 0.33–3.10 | 0.03 | -0.23 | | 30.86 | |
|  | E3 | 1.93 | 2.60 | |  | | 2.02 | | 0.44 | | 0.70–3.80 | 0.18 | 2.62 | | 21.78 | |
|  | E4 | 3.62 | 5.60^**^ | |  | | 3.20 | | 0.53 | | 2.02–5.06 | 0.66 | 0.74 | | 16.56 | |
|  | E5 | 3.26 | 5.09^*^ | |  | | 3.73 | | 0.54 | | 2.08–5.31 | 0.34 | 0.43 | | 14.48 | |
|  | E6 | 2.97 | 3.92 | |  | | 2.77 | | 0.68 | | 1.30–4.57 | 0.25 | -0.18 | | 24.55 | |
|  | E7 | 2.30 | 3.17 | |  | | 2.56 | | 0.74 | | 0.80–4.93 | 0.19 | 1.37 | | 28.91 | |
|  | E8 | 3.00 | 3.67 | |  | | 3.49 | | 0.73 | | 1.80–6.13 | 0.72 | 0.99 | | 20.92 | |
| FSN | E1 | 14.25 | 15.85^*^ | |  | | 14.62 | | 0.87 | | 11.80–17.20 | 0.14 | 0.19 | | 5.95 | |
|  | E2 | 14.40 | 16.65 | |  | | 15.43 | | 1.12 | | 11.88–17.50 | -0.86 | 1.08 | | 7.26 | |
|  | E3 | 15.46 | 17.50^*^ | |  | | 15.21 | | 0.82 | | 11.65–17.00 | -0.83 | 1.81 | | 5.39 | |
|  | E4 | 15.26 | 16.85^*^ | |  | | 15.04 | | 0.66 | | 13.4–17.37 | 0.50 | 0.92 | | 4.39 | |
|  | E5 | 14.69 | 16.76^*^ | |  | | 15.09 | | 0.61 | | 13.33–16.47 | 0.09 | -0.13 | | 4.04 | |
|  | E6 | 15.34 | 16.26 | |  | | 14.76 | | 0.71 | | 12.92–16.77 | -0.15 | 0.17 | | 4.81 | |
|  | E7 | 14.86 | 17.09 | |  | | 14.60 | | 0.79 | | 12.25–17.00 | -0.05 | 0.45 | | 5.41 | |
|  | E8 | 17.29 | 18.42 | |  | | 16.95 | | 0.72 | | 15.41–19.28 | 0.30 | 0.01 | | 4.25 | |
| GWP | E1 | 4.66 | 5.72 | |  | | 4.71 | | 1.11 | | 2.18–7.54 | 0.16 | -0.36 | | 23.57 | |
|  | E2 | 2.89 | 3.40 | |  | | 2.90 | | 0.81 | | 0.99–4.98 | 0.21 | -0.17 | | 27.93 | |
|  | E3 | 3.60 | 6.14^*^ | |  | | 3.73 | | 0.77 | | 1.50–6.49 | 0.19 | 1.08 | | 20.64 | |
|  | E4 | 4.85 | 8.45^**^ | |  | | 4.47 | | 0.80 | | 2.25–6.84 | 0.30 | 0.56 | | 17.90 | |
|  | E5 | 5.24 | 9.74^*^ | |  | | 7.32 | | 1.24 | | 4.39–11.61 | 0.22 | 0.41 | | 16.94 | |
|  | E6 | 4.89 | 6.37 | |  | | 4.56 | | 1.17 | | 2.41–8.44 | 0.51 | 0.26 | | 25.66 | |
|  | E7 | 4.43 | 6.35 | |  | | 4.61 | | 1.26 | | 1.96–9.77 | 0.87 | 1.96 | | 27.33 | |
|  | E8 | 6.00 | 6.83 | |  | | 6.32 | | 1.65 | | 2.37–12.16 | 0.49 | 0.38 | | 26.11 | |

PH, plant height; SL, spike length; NT, the number of valid tillers; FSN, fertile spikelet number per main spike; GNS, grain number per spike; GWP, grain weight per plant; TGW, thousand-grain weight; HD, heading date; SD, standard deviation; CV, coefficient of variation.

^*^, ^**^ represent the significance at *P*=0.05 and *P*=0.01 levels between Lumai 14 and Shaanhan 8675, respectively, by *t*-tests.
