# Supplementary material for: Mining the stable quantitative trait loci for agronomic traits in wheat (Triticum aestivum L.) based on an introgression line population
Source: BMC Plant Biol. 2020 Jun 15;20:275. doi: 10.1186/s12870-020-02488-z (PMC7296640; doi:10.1186/s12870-020-02488-z)
Supplement: Supplementary file 2 — Additional file 2. Plant height characteristics in wheat lines carrying introgressed donor chromosomal segments at the qPH-6A locus. [file 12870_2020_2488_MOESM2_ESM.docx]

**Additional file 2** Plant height characteristics in wheat lines carrying introgressed donor chromosomal segments at the *qPH-6A* locus

| Line | Number of introgressed segments | Introgressed QTL for plant height | Additive effect of QTL | Plant height (cm) | | | | | | | |
| --- | --- | --- | --- | --- | --- | --- | --- | --- | --- | --- | --- |
|  |  |  |  | E1 | E2 | E3 | E4 | E5 | E6 | E7 | E8 |
| 1 | 14 | *qPH-6A* | ＋ | 61.14^**^ | 61.63^**^ | 67.50^**^ | 65.73 | 87.75^**^ | 56.95^*^ | 58.28 | 58.76 |
| 21 | 7 | *qPH-6A* | ＋ | 52.41 | 55.83 | 50.95 | 64.78 | 87.52^**^ | 56.82^*^ | 52.36 | 58.81 |
| 22 | 6 | *qPH-6A* | ＋ | 52.00 | 52.87 | 42.60^**^ | 65.57 | 86.97^**^ | 54.87 | 57.87 | 58.43 |
| 23 | 4 | *qPH-6A* | ＋ | 48.40^**^ | 50.91 | 38.13^**^ | 62.43 | 75.11 | 49.79 | 49.88 | 55.88 |
| 24 | 7 | *qPH-6A* | ＋ | 54.44 | 50.49 | 51.65 | 61.65 | 80.12 | 56.79^*^ | 55.35 | 58.11 |
| 57 | 7 | *qPH-6A* | ＋ | 54.39 | 55.95 | 53.40 | 63.05 | 85.11^*^ | 54.16 | 56.31 | 57.78 |
| 58 | 7 | *qPH-6A* | ＋ | 53.29 | 53.36 | 56.15 | 63.59 | 83.06 | 57.81^**^ | 56.27 | 62.86 |
| 86 | 30 | *qPH-6A* | ＋ | 58.63^**^ | 53.85 | 55.00 | 65.58 | 84.37^*^ | 52.11 | 53.78 | 52.03^*^ |
| 88 | 7 | *qPH-6A* | ＋ | 59.67^**^ | 56.76 | 59.75 | 64.09 | 81.55 | 56.19^*^ | 60.60 | 58.30 |
| 89 | 7 | *qPH-6A* | ＋ | 65.79^**^ | 56.64 | 62.30 | 63.88 | 85.25^*^ | 54.79 | 57.22 | 59.92 |
| 104 | 5 | *qPH-6A* | ＋ | 59.23^**^ | 51.30 | 57.95 | 64.73 | 78.13 | 52.05 | 55.40 | 59.06 |
| 118 | 5 | *qPH-6A* | ＋ | 62.89^**^ | 54.93 | 57.75 | 66.77 | 82.19 | 57.52^*^ | 58.16 | 61.73 |
| 125 | 7 | *qPH-6A* | ＋ | 54.13 | 52.99 | 57.45 | 67.97^*^ | 84.47^*^ | 52.69 | 56.78 | 57.92 |
| 126 | 5 | *qPH-6A* | ＋ | 57.37^*^ | 55.87 | 58.65 | 69.57^**^ | 87.73^**^ | 57.92^**^ | 60.34 | 65.18 |
| 127 | 4 | *qPH-6A* | ＋ | 50.73 | 49.65 | 53.45 | 61.40 | 75.67 | 52.87 | 52.13 | 55.73 |
| 128 | 9 | *qPH-6A* | ＋ | 63.72^**^ | 55.57 | 57.50 | 66.39 | 87.10^**^ | 56.99^*^ | 58.18 | 61.79 |
| 139 | 7 | *qPH-6A* | ＋ | 58.60^**^ | 53.89 | 56.55 | 63.25 | 78.83 | 56.57^*^ | 55.35 | 61.95 |
| 140 | 7 | *qPH-6A* | ＋ | 62.31^**^ | 53.03 | 55.35 | 63.53 | 78.83 | 56.11 | 50.84 | 63.47 |
| 141 | 6 | *qPH-6A* | ＋ | 60.41^**^ | 54.35 | 56.55 | 66.25 | 79.65 | 58.27^**^ | 56.96 | 65.88 |
| 147 | 10 | *qPH-6A* | ＋ | 66.85^**^ | 54.95 | 58.70 | 65.79 | 82.00 | 60.57^**^ | 59.21 | 66.86 |
| 157 | 5 | *qPH-6A* | ＋ | 54.21 | 51.40 | 56.35 | 63.42 | 77.00 | 53.03 | 54.91 | 67.75^*^ |
| 3 | 11 | *qPH-6A, qPH-1B* | ＋ － | 57.66^*^ | 47.54^*^ | 54.45 | 62.41 | 79.78 | 57.96^**^ | 52.05 | 62.56 |
| 42 | 10 | *qPH-6A, qPH-4A* | ＋ － | 59.88^**^ | 49.50 | 53.00 | 61.25 | 78.11 | 53.83 | 51.83 | 56.16 |
| 72 | 18 | *qPH-6A, qPH-1B, qPH-2A, qPH-4A* | ＋ － － － | 54.02 | 51.83 | 56.00 | 61.42 | 79.49 | 52.45 | 54.35 | 55.46 |
| 91 | 7 | *qPH-6A, qPH-1B* | ＋ － | 58.41^**^ | 57.85^*^ | 59.30 | 65.29 | 81.77 | 55.69 | 59.31 | 58.77 |
| 100 | 10 | *qPH-6A, qPH-1B* | ＋ － | 58.77^**^ | 51.91 | 58.05 | 60.51 | 79.95 | 55.29 | 59.41 | 56.55 |
| 101 | 26 | *qPH-6A, qPH-1B, qPH-2A* | ＋ － － | 60.03^**^ | 50.92 | 58.25 | 60.77 | 75.93 | 52.17 | 53.79 | 54.84 |
| 102 | 11 | *qPH-6A, qPH-4A* | ＋ － | 60.17^**^ | 57.64^*^ | 58.80 | 59.65 | 79.43 | 56.31^*^ | 56.04 | 57.42 |
| 108 | 5 | *qPH-6A, qPH-1B* | ＋ － | 61.40^**^ | 54.65 | 56.10 | 66.49 | 88.85^**^ | 55.63 | 59.55 | 62.40 |
| 110 | 6 | *qPH-6A, qPH-1B* | ＋ － | 55.42 | 50.72 | 54.60 | 59.59 | 75.63 | 52.77 | 56.51 | 56.09 |
| 117 | 11 | *qPH-6A, qPH-1B* | ＋ － | 63.96^**^ | 54.01 | 57.50 | 66.34 | 83.25 | 55.41 | 58.33 | 59.53 |
| 136 | 12 | *qPH-6A, qPH-1B, qPH-2A* | ＋ － － | 71.71^**^ | 57.16 | 64.75^**^ | 79.12^**^ | 100.01^**^ | 60.38^**^ | 66.33^**^ | 71.40^**^ |
| 142 | 8 | *qPH-6A, qPH-1B* | ＋ － | 56.43 | 50.87 | 50.50^*^ | 71.17^**^ | 94.29^**^ | 62.63^**^ | 60.01 | 67.35^*^ |
| 145 | 9 | *qPH-6A, qPH-4A* | ＋ － | 63.70^**^ | 50.93 | 55.50 | 60.94 | 78.92 | 55.65 | 55.19 | 65.12 |
| 146 | 10 | *qPH-6A, qPH-1B, qPH-2A, qPH-4A* | ＋ － － － | 61.31^**^ | 50.51 | 55.25 | 63.54 | 76.05 | 53.95 | 53.93 | 61.45 |
| 149 | 14 | *qPH-6A, qPH-1B* | ＋ － | 65.36^**^ | 54.91 | 60.60 | 69.92^**^ | 84.55^*^ | 62.15^**^ | 66.15^**^ | 67.21^*^ |
| 151 | 8 | *qPH-6A, qPH-1B* | ＋ － | 65.85^**^ | 56.03 | 57.05 | 66.72 | 80.33 | 61.29^**^ | 58.56 | 77.45^**^ |
| 152 | 9 | *qPH-6A, qPH-3A, qPH-4A* | ＋ ＋ － | 70.23^**^ | 54.18 | 61.41 | 70.61^**^ | 77.34 | 59.52^**^ | 57.71 | 79.47^**^ |
| Lumai 14 | |  |  | 53.76 | 52.65 | 56.38 | 60.98 | 77.27 | 51.83 | 52.43 | 59.83 |

Positive “additive effect” indicates an increasing effect from ‘Shaanhan 8675’; negative “additive effect” indicates an increasing effect from ‘Lumai 14’.

^*^, ^**^ represent the significance at *P*=0.05 and *P*=0.01 levels between ILs and Lumai 14, respectively, by LSD-*t* tests.
