# Supplementary material for: Mining the stable quantitative trait loci for agronomic traits in wheat (Triticum aestivum L.) based on an introgression line population
Source: BMC Plant Biol. 2020 Jun 15;20:275. doi: 10.1186/s12870-020-02488-z (PMC7296640; doi:10.1186/s12870-020-02488-z)
Supplement: Supplementary file 3 — Additional file 3. Spike length characteristics in wheat lines carrying introgressed donor chromosomal segments at the qSL-2A locus. [file 12870_2020_2488_MOESM3_ESM.docx]

**Additional file 3** Spike length characteristics in wheat lines carrying introgressed donor chromosomal segments at the *qSL-2A* locus

| Line | Number of introgressed segments | Introgressed QTL for spike length | Additive effect of QTL | Spike length (cm) | | | | | | | |
| --- | --- | --- | --- | --- | --- | --- | --- | --- | --- | --- | --- |
|  |  |  |  | E1 | E2 | E3 | E4 | E5 | E6 | E7 | E8 |
| 18 | 11 | *qSL-2A* | － | 6.68^**^ | 7.82 | 7.45^**^ | 7.91^**^ | 8.35^*^ | 8.31 | 8.39 | 7.13^**^ |
| 39 | 7 | *qSL-2A* | － | 7.56 | 7.95 | 7.10^**^ | 8.05^**^ | 7.75^**^ | 7.13^**^ | 7.66^*^ | 6.49^**^ |
| 40 | 10 | *qSL-2A* | － | 7.59 | 8.19 | 8.15 | 8.41 | 8.79 | 8.34 | 8.59 | 7.65 |
| 5 | 10 | *qSL-2A,qSL-7D* | － ＋ | 7.79 | 7.68 | 8.25 | 8.95 | 9.33 | 9.00 | 8.47 | 7.71 |
| 17 | 15 | *qSL-2A,qSL-1B* | － － | 6.93^**^ | 6.91^**^ | 7.20^**^ | 7.51^**^ | 8.48^*^ | 7.36^**^ | 7.23^**^ | 6.93^**^ |
| 45 | 46 | *qSL-2A,qSL-1B,qSL-2B,qSL-7A-1,qSL-7A-2* | － －＋ ＋ ＋ | 8.88^**^ | 9.69^**^ | 9.10 | 9.64^*^ | 10.67^**^ | 9.33 | 9.14^*^ | 8.15 |
| 53 | 14 | *qSL-2A,qSL-1B,qSL-7D* | － －＋ | 6.87^**^ | 7.01^**^ | 7.10^**^ | 7.55^**^ | 7.83^**^ | 7.25^**^ | 7.29^**^ | 6.81^**^ |
| 54 | 10 | *qSL-2A,qSL-7D* | －＋ | 7.79 | 7.68 | 8.10 | 8.85 | 9.02 | 8.55 | 8.69 | 7.82 |
| 55 | 7 | *qSL-2A,qSL-7D* | －＋ | 7.52 | 8.28 | 7.60^*^ | 8.41 | 8.77 | 8.34 | 8.30 | 7.47^*^ |
| 70 | 7 | *qSL-2A,qSL-1A* | － ＋ | 7.32 | 8.20 | 7.95 | 8.33 | 8.87 | 8.51 | 8.67 | 7.23^**^ |
| 72 | 18 | *qSL-2A,qSL-1A,qSL-1B,qSL-6A,qSL-7D* | － ＋ －＋ ＋ | 7.94^*^ | 7.77 | 7.85 | 8.23^*^ | 8.41^*^ | 8.23 | 8.27 | 7.24^**^ |
| 86 | 30 | *qSL-2A,qSL-1A,qSL-6A* | － ＋ ＋ | 8.00^*^ | 8.30 | 8.15 | 9.21 | 8.45^*^ | 7.88^**^ | 8.10 | 7.33^*^ |
| 93 | 12 | *qSL-2A,qSL-1A,qSL-7D* | － ＋ ＋ | 9.13^**^ | 9.47^**^ | 8.45 | 9.71^**^ | 10.12^*^ | 8.78 | 9.41^**^ | 8.71 |
| 101 | 26 | *qSL-2A,qSL-1A,qSL-1B,qSL-6A* | － ＋ －＋ | 7.80 | 8.16 | 8.10 | 8.47 | 8.23^**^ | 8.19 | 8.21 | 7.94 |
| 106 | 8 | *qSL-2A,qSL-1B* | － － | 6.22^**^ | 6.63^**^ | 7.15^**^ | 7.17^**^ | 7.63^**^ | 7.01^**^ | 8.46 | 6.45^**^ |
| 121 | 10 | *qSL-2A,qSL-1B* | － － | 7.02^*^ | 7.67 | 7.50^**^ | 8.77 | 8.45^*^ | 8.27 | 8.13 | 7.51^*^ |
| 122 | 8 | *qSL-2A,qSL-1B* | － － | 7.81 | 7.38^*^ | 8.05 | 8.39 | 8.69 | 8.42 | 7.97 | 7.75 |
| 131 | 10 | *qSL-2A,qSL-1B* | － － | 6.52^**^ | 7.89 | 7.45^**^ | 8.33 | 8.33^*^ | 8.08^*^ | 8.29 | 7.50^*^ |
| 136 | 12 | *qSL-2A,qSL-1B,qSL-6A* | － －＋ | 7.41 | 7.84 | 8.05 | 8.63 | 8.74 | 8.16 | 8.62 | 7.76 |
| 137 | 9 | *qSL-2A,qSL-1B* | － － | 6.45^**^ | 7.48 | 6.55^**^ | 7.53^**^ | 7.61^**^ | 7.34^**^ | 7.40^**^ | 6.57^**^ |
| 143 | 43 | *qSL-2A,qSL-1A,qSL-1B,qSL-7D* | － ＋ －＋ | 8.23^**^ | 8.00 | 7.70^*^ | 9.21 | 7.98^**^ | 7.93^*^ | 7.79^*^ | 8.20 |
| 146 | 10 | *qSL-2A,qSL-1B,qSL-6A* | － －＋ | 7.27 | 7.51 | 7.20^**^ | 8.14^*^ | 8.31^*^ | 7.69^**^ | 7.73^*^ | 7.33^*^ |
| 153 | 12 | *qSL-2A,qSL-1A,qSL-1B,qSL-7D* | － ＋ －＋ | 7.77 | 7.85 | 7.45^**^ | 8.93 | 8.69 | 8.56 | 8.48 | 7.80 |
| Lumai 14 |  |  |  | 7.54 | 8.21 | 8.52 | 8.90 | 9.09 | 8.81 | 8.50 | 8.24 |

Positive “additive effect” indicates an increasing effect from ‘Shaanhan 8675’; negative “additive effect” indicates an increasing effect from ‘Lumai 14’.

^*^, ^**^ represent the significance at *P*=0.05 and *P*=0.01 levels between ILs and Lumai 14, respectively, by LSD-*t* tests.
