# Supplementary material for: Mining the stable quantitative trait loci for agronomic traits in wheat (Triticum aestivum L.) based on an introgression line population
Source: BMC Plant Biol. 2020 Jun 15;20:275. doi: 10.1186/s12870-020-02488-z (PMC7296640; doi:10.1186/s12870-020-02488-z)
Supplement: Supplementary file 4 — Additional file 4. Heading date characteristics in wheat lines carrying introgressed donor chromosomal segments at the qHD-1A locus. [file 12870_2020_2488_MOESM4_ESM.docx]

**Additional file 4** Heading date characteristics in wheat lines carrying introgressed donor chromosomal segments at the *qHD-1A* locus

| Line | Number of introgressed segments | Introgressed QTL for heading date | Additive effect of QTL | Heading date (days) | | | | | | | |
| --- | --- | --- | --- | --- | --- | --- | --- | --- | --- | --- | --- |
|  |  |  |  | E1 | E2 | E3 | E4 | E5 | E6 | E7 | E8 |
| 15 | 11 | *qHD-1A* | － | 222.00^*^ | 223.00 | 226.00^*^ | 214.67 | 216.00^**^ | 215.00^*^ | 220.00 | 221.33^*^ |
| 16 | 13 | *qHD-1A* | － | 223.33 | 222.33^*^ | 224.00 | 214.33 | 216.67^**^ | 215.33 | 218.33 | 221.33^*^ |
| 29 | 8 | *qHD-1A* | － | 224.00 | 223.00 | 224.00 | 214.33 | 215.33^**^ | 214.00^**^ | 218.33 | 221.33^*^ |
| 35 | 9 | *qHD-1A* | － | 224.00 | 223.33 | 220.00^*^ | 215.67 | 217.00^*^ | 215.33 | 220.00 | 221.67 |
| 96 | 8 | *qHD-1A* | － | 224.33 | 222.33^*^ | 223.00 | 216.00 | 215.33^**^ | 214.50^**^ | 218.33 | 221.67 |
| 97 | 9 | *qHD-1A* | － | 224.33 | 223.33 | 222.33 | 215.00 | 216.00^**^ | 215.33 | 218.67 | 220.67^**^ |
| 98 | 10 | *qHD-1A* | － | 225.00 | 222.00^**^ | 222.33 | 213.67^*^ | 216.33^**^ | 214.33^**^ | 218.00 | 221.00^**^ |
| 102 | 11 | *qHD-1A* | － | 225.33 | 224.00 | 222.00 | 215.33 | 216.00^**^ | 214.33^**^ | 218.67 | 221.00^**^ |
| 119 | 5 | *qHD-1A* | － | 224.00 | 223.00 | 224.33 | 215.00 | 216.33^**^ | 215.00^*^ | 218.33 | 222.00 |
| 120 | 8 | *qHD-1A* | － | 224.33 | 224.00 | 222.33 | 215.00 | 216.00^**^ | 214.33^**^ | 217.67 | 222.67 |
| 128 | 9 | *qHD-1A* | － | 224.33 | 222.33^*^ | 225.00 | 214.67 | 216.00^**^ | 215.33 | 217.67 | 221.00^**^ |
| 27 | 8 | *qHD-1A,qHD-2D* | － － | 222.00^*^ | 223.33 | 223.33 | 214.67 | 214.67^**^ | 214.33^**^ | 218.00 | 222.00 |
| 28 | 14 | *qHD-1A,qHD-2D* | － － | 222.33^*^ | 223.33 | 223.00 | 213.67^*^ | 215.33^**^ | 213.67^**^ | 217.67 | 221.00^**^ |
| 32 | 9 | *qHD-1A,qHD-1B* | －＋ | 224.00 | 222.33^*^ | 223.00 | 214.33 | 216.00^**^ | 214.67^*^ | 217.67 | 221.33^*^ |
| 45 | 46 | *qHD-1A,qHD-1B,qHD-6B* | －＋＋ | 223.00 | 224.33 | 224.00 | 215.33 | 216.00^**^ | 215.33 | 219.00 | 222.00 |
| 61 | 7 | *qHD-1A,qHD-1B,qHD-2D* | －＋ － | 222.33^*^ | 222.33^*^ | 223.00 | 216.33 | 214.67^**^ | 213.33^**^ | 217.00^*^ | 220.33^**^ |
| 62 | 7 | *qHD-1A,qHD-2D* | － － | 224.33 | 222.33^*^ | 222.00 | 214.00 | 214.67^**^ | 213.67^**^ | 217.33^*^ | 221.00^**^ |
| 63 | 7 | *qHD-1A,qHD-1B* | －＋ | 225.33 | 223.00 | 224.33 | 216.67 | 216.67^**^ | 215.00^*^ | 218.00 | 220.00^**^ |
| 64 | 6 | *qHD-1A,qHD-1B* | －＋ | 224.33 | 223.00 | 224.33 | 218.00 | 216.33^**^ | 214.67^*^ | 218.33 | 221.00^**^ |
| 143 | 43 | *qHD-1A,qHD-1B,qHD-6B* | －＋＋ | 223.00 | 223.33 | 224.33 | 216.00 | 216.00^**^ | 216.33 | 221.00 | 221.33^*^ |
| Lumai 14 |  |  |  | 224.84 | 224.17 | 223.00 | 216.00 | 218.33 | 216.11 | 219.67 | 222.78 |

Positive “additive effect” indicates an increasing effect from ‘Shaanhan 8675’; negative “additive effect” indicates an increasing effect from ‘Lumai 14’.

^*^, ^**^ represent the significance at *P*=0.05 and *P*=0.01 levels between ILs and Lumai 14, respectively, by LSD-*t* tests.
