# Supplementary material for: Mining the stable quantitative trait loci for agronomic traits in wheat (Triticum aestivum L.) based on an introgression line population
Source: BMC Plant Biol. 2020 Jun 15;20:275. doi: 10.1186/s12870-020-02488-z (PMC7296640; doi:10.1186/s12870-020-02488-z)
Supplement: Supplementary file 5 — Additional file 5. Heading date characteristics in wheat lines carrying introgressed donor chromosomal segments at the qHD-2D locus. [file 12870_2020_2488_MOESM5_ESM.docx]

**Additional file** 5 Heading date characteristics in wheat lines carrying introgressed donor chromosomal segments at the *qHD-2D* locus

| Line | Number of introgressed segments | Introgressed QTL for heading date | Additive effect of QTL | heading date (days) | | | | | | | |
| --- | --- | --- | --- | --- | --- | --- | --- | --- | --- | --- | --- |
|  |  |  |  | E1 | E2 | E3 | E4 | E5 | E6 | E7 | E8 |
| 49 | 6 | *qHD-2D* | － | 224.33 | 224.33 | 224.00 | 215.67 | 216.67^*^ | 215.00^*^ | 219.00 | 221.00^**^ |
| 50 | 4 | *qHD-2D* | － | 224.00 | 224.00 | 223.00 | 216.33 | 217.33 | 214.33^**^ | 218.00^*^ | 221.00^**^ |
| 66 | 7 | *qHD-2D* | － | 223.33 | 224.00 | 223.00 | 215.67 | 217.33 | 215.33 | 217.67^**^ | 221.00^**^ |
| 84 | 8 | *qHD-2D* | － | 226.00 | 223.00 | 222.33 | 215.67 | 216.67^*^ | 214.33^**^ | 216.33^**^ | 221.33 |
| 87 | 14 | *qHD-2D* | － | 224.33 | 224.33 | 223.33 | 216.67 | 217.33 | 214.67^**^ | 217.00^**^ | 221.67 |
| 105 | 5 | *qHD-2D* | － | 224.33 | 222.33^**^ | 223.00 | 216.33 | 216.00^**^ | 214.67^**^ | 218.33 | 221.67 |
| 145 | 9 | *qHD-2D* | － | 225.33 | 224.33 | 222.33 | 216.67 | 216.33^**^ | 215.67 | 218.33 | 221.00^**^ |
| 27 | 8 | *qHD-2D,qHD-1A* | － － | 222.00^**^ | 223.33 | 223.33 | 214.67 | 214.67^**^ | 214.33^**^ | 218.00^*^ | 222.00 |
| 28 | 14 | *qHD-2D,qHD-1A* | － － | 222.33^*^ | 223.33 | 223.00 | 213.67^*^ | 215.33^**^ | 213.67^**^ | 217.67^**^ | 221.00^**^ |
| 53 | 14 | *qHD-2D,qHD-1B* | －＋ | 226.00 | 224.00 | 222.33 | 217.33 | 216.33^**^ | 216.00 | 219.00 | 221.67 |
| 61 | 7 | *qHD-2D,qHD-1A,qHD-1B* | － －＋ | 222.33^*^ | 222.33^**^ | 223.00 | 216.33 | 214.67^**^ | 213.33^**^ | 217.00^**^ | 220.33^**^ |
| 62 | 7 | *qHD-2D,qHD-1A* | － － | 224.33 | 222.33^**^ | 222.00 | 214.00* | 214.67^**^ | 213.67^**^ | 217.33^**^ | 221.00^**^ |
| 65 | 9 | *qHD-2D,qHD-1B* | －＋ | 224.00 | 223.33 | 223.00 | 214.33 | 216.00^**^ | 214.00^**^ | 217.00^**^ | 220.67^**^ |
| 113 | 7 | *qHD-2D,qHD-1B* | －＋ | 225.00 | 224.00 | 223.00 | 215.67 | 217.67 | 215.33 | 218.67 | 221.67 |
| 114 | 7 | *qHD-2D,qHD-1B* | －＋ | 225.33 | 224.00 | 223.33 | 215.67 | 217.00^*^ | 215.33 | 219.00 | 221.67 |
| 142 | 8 | *qHD-2D,qHD-1B* | －＋ | 223.33 | 224.00 | 224.00 | 216.67 | 216.00^**^ | 215.00^*^ | 219.67 | 221.33 |
| 148 | 7 | *qHD-2D,qHD-1B* | －＋ | 224.33 | 223.33 | 223.00 | 214.33 | 216.33^**^ | 215.33 | 218.33 | 222.00 |
| 149 | 14 | *qHD-2D,qHD-1B* | －＋ | 223.33 | 224.33 | 223.33 | 214.67 | 217.00^*^ | 214.67^**^ | 217.33^**^ | 222.00 |
| Lumai 14 |  |  |  | 224.84 | 224.17 | 223.00 | 216.00 | 218.33 | 216.11 | 219.67 | 222.78 |

Positive “additive effect” indicates an increasing effect from ‘Shaanhan 8675’; negative “additive effect” indicates an increasing effect from ‘Lumai 14’.

^*^, ^**^ represent the significance at *P*=0.05 and *P*=0.01 levels between ILs and Lumai 14, respectively, by LSD-*t* tests.
