# Supplementary material for: Mining the stable quantitative trait loci for agronomic traits in wheat (Triticum aestivum L.) based on an introgression line population
Source: BMC Plant Biol. 2020 Jun 15;20:275. doi: 10.1186/s12870-020-02488-z (PMC7296640; doi:10.1186/s12870-020-02488-z)
Supplement: Supplementary file 6 — Additional file 6. Spike length characteristics in wheat lines carrying introgressed donor chromosomal segments at the qSL-6A locus. [file 12870_2020_2488_MOESM6_ESM.docx]

**Additional file6** Spike length characteristics in wheat lines carrying introgressed donor chromosomal segments at the *qSL-6A* locus

| Line | Number of introgressed segments | Introgressed QTL for heading date | Additive effect of QTL | heading date (days) | | | | | | | |
| --- | --- | --- | --- | --- | --- | --- | --- | --- | --- | --- | --- |
|  |  |  |  | E1 | E2 | E3 | E4 | E5 | E6 | E7 | E8 |
| 21 | 7 | *qSL-6A* | ＋ | 7.22 | 8.56 | 7.60^**^ | 8.54 | 9.67 | 8.89 | 8.27 | 7.85 |
| 22 | 6 | *qSL-6A* | ＋ | 7.73 | 8.39 | 7.10^**^ | 9.27 | 9.64 | 8.82 | 9.38^**^ | 8.67 |
| 23 | 4 | *qSL-6A* | ＋ | 7.00^**^ | 7.35^**^ | 6.42^**^ | 8.78 | 8.87 | 7.90^*^ | 7.48^**^ | 7.43^*^ |
| 24 | 7 | *qSL-6A* | ＋ | 8.02^**^ | 7.85 | 7.95^*^ | 9.87^*^ | 9.63 | 9.40 | 8.63 | 8.04 |
| 102 | 11 | *qSL-6A* | ＋ | 7.61 | 9.15^**^ | 9.05^*^ | 8.36 | 9.38 | 8.89 | 8.79 | 8.14 |
| 104 | 5 | *qSL-6A* | ＋ | 8.07^**^ | 8.81^*^ | 8.75 | 9.53 | 9.37 | 8.56 | 8.74 | 9.05^*^ |
| 118 | 5 | *qSL-6A* | ＋ | 8.09^**^ | 8.50 | 8.50 | 8.93 | 9.32 | 9.02 | 9.13^*^ | 8.73 |
| 125 | 7 | *qSL-6A* | ＋ | 7.96^*^ | 7.97 | 7.85^**^ | 9.05 | 9.07 | 8.30 | 8.36 | 8.03 |
| 126 | 5 | *qSL-6A* | ＋ | 8.03^**^ | 8.86^**^ | 8.55 | 9.95^**^ | 9.71 | 9.13 | 9.33^**^ | 9.29^**^ |
| 127 | 4 | *qSL-6A* | ＋ | 6.80^**^ | 7.69^*^ | 7.60^**^ | 8.13^*^ | 8.42 | 8.21 | 7.85^*^ | 7.56 |
| 128 | 9 | *qSL-6A* | ＋ | 8.50^**^ | 8.66 | 8.65 | 8.48 | 8.39 | 9.24 | 8.67 | 8.39 |
| 139 | 7 | *qSL-6A* | ＋ | 8.03^**^ | 8.14 | 7.70^**^ | 9.50 | 9.93^*^ | 9.15 | 8.58 | 8.57 |
| 140 | 7 | *qSL-6A* | ＋ | 8.67^**^ | 8.20 | 8.30 | 9.34 | 9.15 | 8.98 | 8.75 | 8.87 |
| 141 | 6 | *qSL-6A* | ＋ | 8.83^**^ | 8.62 | 8.55 | 9.92^**^ | 9.45 | 9.92^**^ | 9.24^*^ | 9.13^*^ |
| 145 | 9 | *qSL-6A* | ＋ | 8.35^**^ | 8.14 | 8.05 | 9.45 | 9.35 | 9.26 | 8.89 | 9.01^*^ |
| 147 | 10 | *qSL-6A* | ＋ | 8.61^**^ | 8.43 | 8.20 | 9.11 | 9.50 | 9.32 | 8.64 | 8.79 |
| 3 | 11 | *qSL-6A, qSL-1B* | ＋ － | 7.53 | 8.01 | 8.50 | 9.19 | 8.97 | 8.85 | 8.35 | 8.13 |
| 42 | 10 | *qSL-6A, qSL-7D* | ＋＋ | 9.24^**^ | 9.83^**^ | 8.85 | 9.37 | 10.23^**^ | 9.81^**^ | 9.47^**^ | 9.23^**^ |
| 57 | 7 | *qSL-6A, qSL-1A* | ＋＋ | 7.68 | 9.41^**^ | 8.45 | 9.51 | 9.39 | 8.88 | 8.76 | 8.93 |
| 58 | 7 | *qSL-6A, qSL-1A* | ＋＋ | 8.34^**^ | 8.33 | 8.50 | 9.50 | 9.47 | 9.19 | 8.53 | 8.71 |
| 88 | 7 | *qSL-6A, qSL-1A* | ＋＋ | 7.55 | 8.78^*^ | 8.85 | 9.31 | 9.79 | 8.90 | 8.91 | 7.97 |
| 89 | 7 | *qSL-6A, qSL-1A* | ＋＋ | 8.68^**^ | 8.78^*^ | 9.00^*^ | 9.37 | 9.83 | 8.57 | 8.85 | 8.09 |
| 108 | 5 | *qSL-6A, qSL-1B* | ＋ － | 7.08^*^ | 7.32^**^ | 7.80^**^ | 8.53 | 8.75 | 8.75 | 8.27 | 8.13 |
| 110 | 6 | *qSL-6A, qSL-1B* | ＋ － | 8.54^**^ | 8.44 | 8.55 | 9.31 | 9.44 | 8.45 | 9.45^**^ | 8.39 |
| 142 | 8 | *qSL-6A, qSL-1B* | ＋ － | 8.07^**^ | 8.50 | 8.15 | 9.89^*^ | 8.67 | 8.55 | 8.49 | 8.41^*^ |
| 149 | 14 | *qSL-6A, qSL-1B* | ＋ － | 8.59^**^ | 8.35 | 8.20 | 9.50 | 9.03 | 9.75^*^ | 9.31^*^ | 9.11^*^ |
| 152 | 9 | *qSL-6A, qSL-7D* | ＋＋ | 9.43^**^ | 9.29^**^ | 9.80^**^ | 11.05^**^ | 10.83^**^ | 10.27^**^ | 9.79^**^ | 10.21^**^ |
| 1 | 14 | *qSL-6A, qSL-7A-1, qSL-7A-2* | ＋＋＋ | 9.51^**^ | 9.29^**^ | 9.65^**^ | 8.46 | 8.63 | 8.15 | 8.06 | 7.29^*^ |
| 86 | 30 | *qSL-6A, qSL-1A, qSL-2A* | ＋＋ － | 8.00^*^ | 8.30 | 8.15 | 9.21 | 8.45 | 7.88^*^ | 8.10 | 7.33^*^ |
| 91 | 7 | *qSL-6A, qSL-1A, qSL-1B* | ＋＋ － | 8.48^**^ | 9.60^**^ | 9.50^**^ | 10.06^**^ | 10.03^*^ | 9.75^*^ | 9.65^**^ | 9.05^*^ |
| 117 | 11 | *qSL-6A, qSL-1A, qSL-1B* | ＋ － | 8.51^**^ | 8.57 | 8.30 | 9.07 | 9.73 | 8.61 | 8.49 | 8.47 |
| 136 | 12 | *qSL-6A, qSL-1B, qSL-2A* | ＋ － － | 7.41 | 7.84 | 8.05 | 8.63 | 8.74 | 8.16 | 8.62 | 7.76 |
| 146 | 10 | *qSL-6A, qSL-1B, qSL-2A* | ＋ － － | 7.27 | 7.51^**^ | 7.20^**^ | 8.14^*^ | 8.31^*^ | 7.69^**^ | 7.73^*^ | 7.33^*^ |
| 157 | 5 | *qSL-6A, qSL-1A, qSL-7D* | ＋＋＋ | 7.66 | 7.77 | 7.95^*^ | 8.42 | 8.88 | 8.23 | 8.62 | 7.94 |
| 100 | 10 | *qSL-6A, qSL-1A, qSL-1B, qSL-7D* | ＋＋ －＋ | 7.94^*^ | 8.81^*^ | 8.30 | 9.06 | 9.63 | 8.57 | 9.58^**^ | 7.73 |
| 101 | 26 | *qSL-6A, qSL-1A, qSL-1B, qSL-2A* | ＋＋ － － | 7.80 | 8.16 | 8.10 | 8.47 | 8.23^*^ | 8.19 | 8.21 | 7.94 |
| 151 | 8 | *qSL-6A, qSL-1A, qSL-1B, qSL-7D* | ＋＋ －＋ | 9.42^**^ | 9.46^**^ | 9.00^*^ | 10.13^**^ | 10.25^**^ | 10.08^**^ | 9.74^**^ | 10.23^**^ |
| 72 | 18 | *qSL-6A, qSL-1A, qSL-1B, qSL-2A, qSL-7D* | ＋＋ － －＋ | 7.94^*^ | 7.77 | 7.85^**^ | 8.23 | 8.41 | 8.23 | 8.27 | 7.24^**^ |
| Lumai 14 |  |  |  | 7.54 | 8.21 | 8.52 | 8.90 | 9.09 | 8.81 | 8.50 | 8.24 |

Positive “additive effect” indicates an increasing effect from ‘Shaanhan 8675’; negative “additive effect” indicates an increasing effect from ‘Lumai 14’.

^*^, ^**^ represent the significance at *P*=0.05 and *P*=0.01 levels between ILs and Lumai 14, respectively, by LSD-*t* tests.
